# Volterra representation enables modeling of complex synaptic nonlinear dynamics in large-scale simulations

**DOI:** 10.3389/fncom.2015.00112

**Published:** 2015-09-17

**Authors:** Eric Y. Hu, Jean-Marie C. Bouteiller, Dong Song, Michel Baudry, Theodore W. Berger

**Affiliations:** ^1^Department of Biomedical Engineering, University of Southern CaliforniaLos Angeles, CA, USA; ^2^Graduate College of Biomedical Sciences, Western University of Health SciencesPomona, CA, USA

**Keywords:** volterra expansion, synaptic modeling, multi-scale modeling, glutamatergic synapse, computational modeling

## Abstract

Chemical synapses are comprised of a wide collection of intricate signaling pathways involving complex dynamics. These mechanisms are often reduced to simple spikes or exponential representations in order to enable computer simulations at higher spatial levels of complexity. However, these representations cannot capture important nonlinear dynamics found in synaptic transmission. Here, we propose an input-output (IO) synapse model capable of generating complex nonlinear dynamics while maintaining low computational complexity. This IO synapse model is an extension of a detailed mechanistic glutamatergic synapse model capable of capturing the input-output relationships of the mechanistic model using the Volterra functional power series. We demonstrate that the IO synapse model is able to successfully track the nonlinear dynamics of the synapse up to the third order with high accuracy. We also evaluate the accuracy of the IO synapse model at different input frequencies and compared its performance with that of kinetic models in compartmental neuron models. Our results demonstrate that the IO synapse model is capable of efficiently replicating complex nonlinear dynamics that were represented in the original mechanistic model and provide a method to replicate complex and diverse synaptic transmission within neuron network simulations.

## Introduction

Computational multi-scale and large-scale modeling are increasingly used to gain insights on brain functions and dysfunctions (Dyhrfjeld-Johnsen et al., 2007; Bouteiller et al., 2011; Hendrickson et al., 2013; Mattioni and Le Novère, 2013; Dougherty et al., 2014; Yu et al., 2014). At the synaptic level, a large number of receptors, mechanisms, and pathways modulate synaptic strength, function, and plasticity. Numerous studies (Sakimura et al., 1995; Nakazawa et al., 2002) have repeatedly shown that modifications of molecular processes can result in functional and behavioral changes. Similarly, many pathological conditions, including autism and Alzheimer's disease, have been hypothesized to have molecular origins (Francis et al., 1999; Trottier et al., 1999; Rogawski, 2013). If computational modeling is to help in better understanding the mechanisms underlying normal and pathological cases, multi- and large-scale models are essential for taking into account relevant processes that take place at all levels.

Mathematical models that simulate physiological systems are developed to depict the system of interest, or at least provide a reasonable view of some of its inherent mechanisms and functions. Markov kinetic state models (MSM) represent a popular choice of model structure used to represent many dynamical physiological systems (Prinz et al., 2011). However, integration of a large number of kinetic models with varying temporal dynamics without simplification can increase the computational loads, ultimately leading to prohibitively long simulation times.

Large networks in the range of 1 million neurons (Izhikevich and Edelman, 2008) and up to 1 billion neurons (Ananthanarayanan et al., 2009) have been successfully modeled with varying amount of detail. These large scale models must adequately address the computational requirements in running simulations with large numbers of models. Current methodologies to accommodate these requirements can be divided into two major strategies: (A) Provide increasing computational power to offset the cost, and (B) reduction of model complexity to improve computational efficiency. In many cases, both strategies are used. For strategy (A), the underlying concept is straightforward: the computational cost of large simulations is met by providing high performance computers, as seen in (Allen et al., 2001; Markram, 2006; Hendrickson et al., 2012). Still, despite significant advances in technology and computing, the computational power required to simulate large-scale models comprising a large number of biomolecular details still exceeds the capabilities of today's most performant computers.

To remedy the issue described above, large-scale models often use empirical representations of the effects originating from smaller-scale systems. This leads us to strategy (B), in which large-scale neuron networks often use simplified models of the smaller-scale neuron and synapse models to alleviate the computational burden. Neuron representations, for example, can include single compartment integrate-and-fire models. Simplified synapse models often consist of alpha synapses (i.e., a basic exponential rise and decay equation) to approximate the waveform of postsynaptic changes in conductance. Many models have been reduced even further, simply limiting the response to Dirac peaks and focus solely on the observation of time-based events. Such simplifications allow each level to be isolated in its own environment with its own set of rules, thereby constraining the overall system complexity. Ultimately, however, using empirical methods to represent microscale parameters overlooks the effects of microscale changes on macroscale systems (Weinan and Engquist, 2003), which thus inherently results in measurable errors between empirical (i.e., approximations) and actual values, particularly with regards to nonlinearity.

This leads to an incentive to instead an create intermediate model, one which does not capture the full details of the Markov kinetic models with all its given states, but instead use an alternate representation to replicate the complex nonlinearities which are lost in more simplistic models such as exponential synapses. In some cases, external modifications were made to the traditional exponential synapse form so that varying non-linearities, including desensitization as well as STDP characteristics like facilitation and depression, may be replicated (Tsodyks et al., 1998; Dittman et al., 2000). These types of models require a priori knowledge on the biological system of interest to calibrate their nonlinear features, which could prove limiting in cases where sources of nonlinear dynamics are not yet fully known. Synapses are very complex structures with many different cellular pathways and mechanisms, much of which is still being investigated; defining all possible nonlinear sources beforehand in a model would be difficult, if not impossible, when the knowledge of these synaptic mechanisms is still incomplete. Currently, the nonlinear synapse models developed so far can only take into account more of the commonly noted nonlinear behaviors. Regardless, they provide an efficient, nonlinear alternative to traditional exponential synapses and have helped demonstrate the impact of nonlinear synapses in neuron population models (Tsodyks et al., 1998).

**Figure 1:**
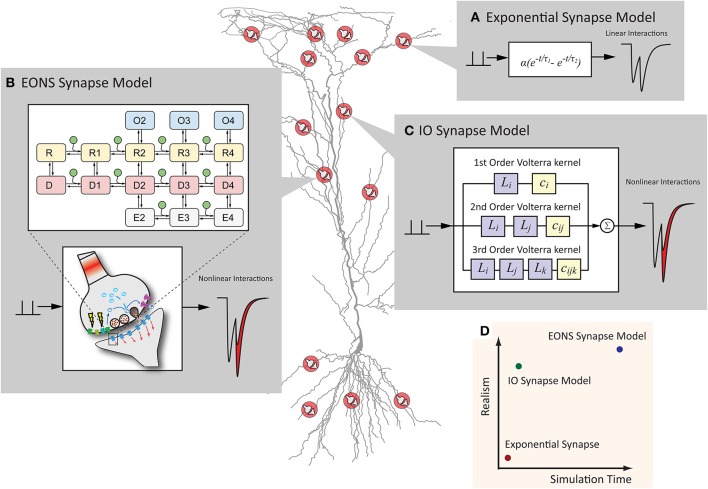
**Synapse models can have various representations, which differ in computational efficiency and model detail. (A)** The exponential synapse is a commonly used synapse model that produces a postsynaptic response from a simplistic equation. The result is fast but lacks more complex dynamics typically seen in an actual synapse. **(B)** The EONS synapse model is a detailed, parametric model of a hippocampal glutamate synapse. Markov kinetic state models and other additional mechanisms govern the overall postsynaptic response, resulting in an accurate and nonlinear response more characteristic of the response that would be observed in an actual glutamatergic synapse. **(C)** The IO synapse model uses the Volterra functional power series to faithfully reproduce the nonlinear details seen in the EONS/RHENOMS model. As this IO synapse model characterizes the dynamic relationships between the input events and the corresponding output, much of the computationally intensive calculations are waived through the use of this methodology. **(D)** Schematic representation of the computation time required and the detailed accuracy of each model. The IO synapse model can provide a much more accurate representation than the exponential synapse, while computationally lighter than the parametric EONS synapse model.

Here, we propose to use the Volterra functional power series (Berger et al., 2010) to capture the dynamics of the nonlinear systems in a very compact form and use them to bridge hierarchical scales (Figure 1). Unlike previous nonlinear synapses, modeling with the Volterra series does not impose any predefined structural assumption on the system it models—the model is instead defined by input-output relations extracted from the data itself, thus making it a functional “input-output” model of the system. The Input-Output (IO) model uses kernels to represent the functional properties of the system modeled, effectively replicating the dynamics and behavior of the process without requiring *a priori* knowledge on its internal structure and its underlying mechanisms. Furthermore, the Volterra-based model requires little computational power. The Input-Output model can therefore reduce complex nonlinear differential systems into input-output transformations, which describe the causal relationship between the input and output properties of the system, while maintaining the nonlinear dynamics of the system model and reducing its computational complexity (Marmarelis and Marmarelis, 1978). The generality of this methodology means that it can be applied to various phenomena, including those from mechanical systems (Bharathy et al., 2008), biomedical systems (Berger et al., 1988a,b; Jo et al., 2007; Song et al., 2009a,b) and economics (Tu et al., 2012).

This article introduces an input-output model of the Elementary Objects of the Nervous System Synaptic Modeling Platform (referred to as the EONS synapse model), using the Volterra functional power series. The EONS synapse model is a detailed, parametric model of the glutamatergic synapse that includes kinetic receptor channels and diffusion mechanisms (Bouteiller et al., 2008). The input-output model, which from this point forward will be referred to as the IO synapse model, is proposed to serve as an extension to the EONS synapse model for multi- and large-scale simulations, and be fitted to the nonlinear dynamics simulated by the EONS synapse model. This article investigates the validity and performance of the IO synapse model through comparisons of the IO synapse model simulation results with the results obtained with the original EONS synapse. To proceed with the validation, first and foremost is a direct comparison between the results of the IO synapse model with EONS in a standalone simulation of the synapse, then simulated alongside a hippocampal CA1 pyramidal cell model in a hybrid simulation environment (Allam et al., 2012). Next, the IO synapse model is provided with random interval train input with mean firing rates ranging from 2 to 10 Hz to determine the degree of nonlinearities that the model is capable of capturing. Finally, to further evaluate the efficiency of the IO synapse model, the kinetic receptor models were implemented directly in the NEURON simulation environment and simulation times between IO and kinetic models were compared. The results clearly indicate that the IO synapse model is capable of replicating the complex functional dynamics of a detailed glutamatergic synapse model, while significantly reducing computational complexity, thereby enabling simulations on larger temporal (seconds to minutes) and spatial scales (large network of neurons containing highly elaborate functional synapses).

## Materials and methods

### Parametric model of the hippocampal CA1 synapse

The synapse model introduced here is a non-parametric representation of the parametric EONS synapse model which simulates a glutamatergic synapse on a CA1 pyramidal cell; details of the model can be found in Bouteiller et al. (2008). Briefly, the EONS synapse model is a highly detailed model of a generic glutamatergic synapse, and includes a number of receptor models as well as various mechanistic properties of the synapse, including but not limited to, voltage-dependent presynaptic calcium entry, probabilistic vesicular release, neurotransmitter diffusion and reuptake, and postsynaptic potential induction through ionotropic receptors. Models used in the EONS synapse model are derived from published experimental results and computational models. Many models in EONS contain differential equations and thus require ODE solvers to compute and simulate. In particular, kinetic models of the ionotropic AMPA and NMDA receptors (noted AMPAr and NMDAr, respectively) include ordinary differential equations requiring a relatively large computation time. The AMPAr model was derived from the model presented in Robert and Howe (2003), and comprises 16 states, each state representing a different conformation of the receptor (open vs. closed, resting or desensitized). The AMPAr current is calculated through the following equation:

$$\begin{array}{l} I_{AMPA}=nb_{AMPA}\times \left(g_{2}O_{2}+g_{3}O_{3}+g_{4}O_{4}\right)\times \left(V-V_{rev}\right) \end{array}$$

where *I*_*AMPA*_I is the total current contributed by AMPAr, O_n_ represent the open states with associated conductances g_n_ with n being the number of glutamate molecules bound, and *V*_*rev*_ is the reversal potential of AMPAr.*nb*_*AMPA*_, which represents the number of AMPA receptors, was set to 80 in the EONS synapse model, which fits the range of approximately 46–174 AMPAr reported in hippocampal synapse (Matsuzaki et al., 2001); AMPAr dynamics are known to be relatively fast (Robert and Howe, 2003), with currents returning to baseline in less than 30 ms. However, the dynamics of AMPAr desensitization are much slower and the receptor can take up to approximately 500 ms to recover from desensitization. The conductance *g*_*AMPA*_ is used for estimations by the IO receptor model of the AMPA receptor model, where:

$$\begin{array}{l} g_{AMPA}=\left(g_{2}O_{2}+g_{3}O_{3}+g_{4}O_{4}\right) \end{array}$$

NMDAr is characterized by slower dynamics than the AMPA receptor (about 300 ms). The kinetic model used in the parametric modeling framework is the 8 states receptor model developed by Erreger et al. (2005) (alternative models were investigated as well—results not presented here). NMDAr current is defined in a similar manner as the AMPAr current:

$$\begin{array}{l} I_{NMDA}=nb_{NMDA}\times g_{NMDA}\times \left(V-V_{rev}\right) \end{array}$$

For NMDAr, the number of NMDA receptors (*nb*_*NMDA*_) is set to 20, which is in range of reported studies (Racca et al., 2000). The parameter *g*_*NMDA*_, however, is intrinsically different from the conductance of AMPAr because of the nonlinear response to voltage due to the magnesium block properties. This feature was separately accounted for in the IO synapse model and is explained in more detail in the next section. Because calculations of both AMPAr and NMDAr kinetic models are time-consuming, constituting a potential bottleneck for larger scale simulations, we propose to derive their corresponding Input-Output counterparts.

### Structure of the input-output model

#### Representation of the overall schematic

**Figure 2:**
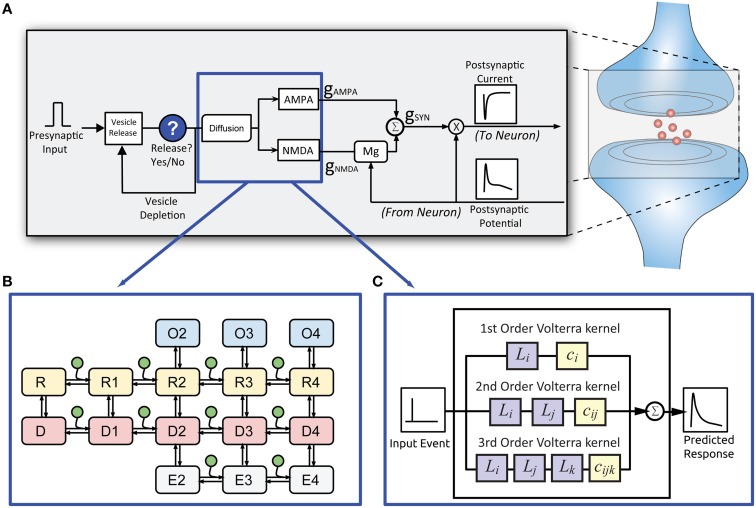
**Structure of the EONS synapse model. (A)** For every presynaptic event, the EONS synapse calculates the probability of vesicle release based on past release events. **(B)** For the original EONS synapse model, in the event of a vesicle release, glutamate diffusion is calculated and depending on postsynaptic receptor location, the result is used for deriving the open states in the kinetic models of the receptors. **(C)** The IO synapse model accounts for both the diffusion and kinetic receptor dynamics to calculate the predicted open states of the receptors. The open states are used to calculate conductances and resulting currents based on the postsynaptic potential. The calculated response is then passed on to the NEURON model.

The framework of the IO synapse model follows a modular structure similar to the original EONS model—see Figure 2 for an overview. The IO synapse model contains three components: the presynaptic release component, the AMPAr component, and the NMDAr component. Using such a modular structure allows for additional components to be easily implemented and integrated in the future. The presynaptic release component uses the Dittman model of facilitation/depression (Dittman et al., 2000), with the parameters described in Song et al. (2009b) to approximate the short term plasticity seen in experimental studies. This model calculates the probability of vesicle release. A random number generator compared with the calculated release probability determines whether a release event occurs or not. If a release event takes place, it is passed on to the AMPAr and NMDAr Input-Output models. The AMPAr IO receptor model calculates the conductance values of AMPAr. For NMDAr, due to the additional complexity of the magnesium block of the channel, the open state probability is calculated first; the receptor conductance is then calculated with the following two equations, as stated in Ambert et al. (2010):

$$\begin{array}{lll} g_{0} & = & g_{1}+\frac{g_{2}-g_{1}}{{1+e}^{\alpha \psi m}}\\ g_{max} & = & \frac{g_{0}}{1+\left(\frac{{Mg}_{0}^{2+}}{K_{0}}\right)e^{-\delta zF}\psi m∕RT}\\ g_{NMDA} & = & g_{max}\times O\left(t\right) \end{array}$$

where *g*_0_ represents the total conductance in the absence of any magnesium, *g*_1_, *g*_2_ represent the open state conductances with one glutamate bound and 2 glutamate molecules bound, respectively. *g*_1_is set at 40 pS while *g*_2_ is set at 247 pS. represents the external magnesium concentration and is set The value α = 0.01 represents the steepness of the transition between *g*_1_ and *g*_2_. $Mg_{0}^{2+}$represents the external magnesium concentration and is set at 1 mM. *K*_0_ is the equilibrium constant for magnesium set at 3.57,*F* is Faraday's Constant (9.64867.104 C mol^−1^), *R* is the molecular gas constant (8.31434 J mol^−1^ K^−1^), and T is the temperature at 273.15 K. The variable ψ*m* represents the affinity between NMDAr and magnesium, which is dependent on the postsynaptic potential of the synapse; the value is set to 0.8. Here we utilize the open state *O*(*t*) as the output data during training of the IO receptor model of the NMDA receptor; the estimated conductance is then calculated from the predicted open state value by the IO receptor model.

#### Reduction of receptor models through the laguerre-volterra modeling

The Input-Output model for receptors uses the Volterra functional power series together with Laguerre basis functions (Berger et al., 2010). The general form of the Volterra functional power series is described by:

$$\begin{array}{lll} u\left(t\right) & = & \;c_{0}+\overset{N}{\underset{n=1}{\sum }}\overset{L}{\underset{j=1}{\sum }}c_{1}^{n}\left(j\right)v_{j}^{n}\left(t\right)\\ +\overset{N}{\underset{n=1}{\sum }}\overset{L}{\underset{j_{1}=1}{\sum }}\overset{j_{1}}{\underset{j_{2}}{\sum }}c_{2s}^{n}\left(j_{1},j_{2}\right)v_{j_{1}}^{n}\left(t\right)v_{j2}^{n}\left(t\right)\\ +\overset{L}{\underset{j_{1}=1}{\sum }}\overset{L}{\underset{j_{2}=1}{\sum }}c_{2x}\left(j_{1},j_{2}\right)v_{j1}^{n_{1}}\left(t\right)v_{j2}^{n_{2}}\left(t\right)\\ v_{j}^{n}\left(t\right) & = & \;\overset{M_{k}}{\underset{\tau =0}{\sum }}b_{j}\left(\tau \right)x_{n}\left(t-\tau \right) \end{array}$$

where *v* represents the basis functions convolved with the input for a memory window length *M*_*k*_ and c_an_ are the scaling coefficients used to have the basis functions fitting the shape of the training data. *N* denotes the number of basis function sets and *L* represents the number of basis functions for each set. Each set is representative of basis functions of different decay constants, which is further elaborated in the description of Laguerre basis functions. Nonlinearities are captured through modeling higher model orders, represented as kernels. For the 0th order kernel, the only required value is c_0_. This corresponds to the baseline signal in the presence of no event. Values for *c*_1_ and *v*_1_ represent the 1st order kernel and account for responses to a single event. *c*_2*s*_ and *v*_2*s*_ represent 2nd order nonlinearities within an individual set of basis functions, whereas *c*_2*x*_ and *v*_2*x*_ represent cross kernels. Nonlinearities occur when multiple events interact with each other and represent the differences between the output of the system and the linear solution of the model given by only the 1st order kernels. As is shown in the equations, 2nd order consists of the basis functions cross multiplying with each other (basis functions within a set are multiplied with each other for 2*s* and basis functions are multiplied with outside sets for 2*x*). Higher orders involve more cross multiplications between basis functions.

For basis functions, the Laguerre equations are used for their orthogonality and convergence properties. Additionally, the signals reproduced using Laguerre basis functions have high resemblance to signals encountered in physiology and biology. More details of the methodology are described in Ghaderi et al. (2011). In brief, the Laguerre basis functions are derived from Laguerre polynomials, which are defined as:

$$\begin{array}{l} L_{n}\left(x\right)=\frac{e^{x}}{n!}\frac{d^{n}}{dx^{n}}\left(-e^{x}x^{n}\right) \end{array}$$

The Laguerre polynomials are orthogonal from the interval of 0 to infinity with a weight function *e*^*x*∕2^, and thus the Laguerre functions can be defined as $l_{n}\left(x\right)=e^{-x∕2}L_{n}\left(x\right)$. In the time domain,−*x*∕2 is replaced with *p*·*t* where *p* is a time scaling factor that corresponds to the decay of the basis functions. With proper normalization the equations take the following form:

$$\{\begin{array}{c} b_{0}\left(t\right)=\;l_{0}\left(t\right)=\sqrt{2p}e^{-pt}\\ b_{1}\left(t\right)=\;l_{1}\left(t\right)=\sqrt{2p}(2pt-1)e^{-pt}\\ b_{2}\left(t\right)=\;l_{2}\left(t\right)=\;\sqrt{2p}(2p^{2}t^{2}-4pt+\;1)e^{-pt}\\ \ldots \end{array}$$

**Figure 3:**
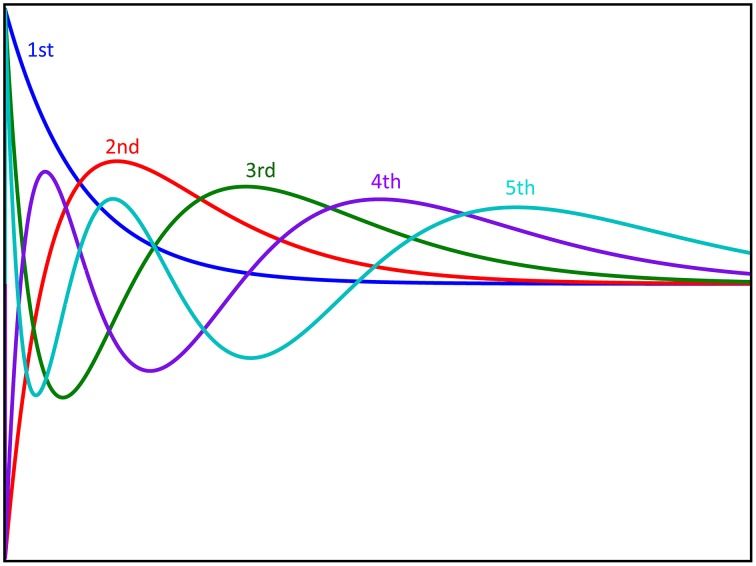
**Graphical representation of the first five Laguerre basis functions**. The basis functions are scaled with coefficients and summed to produce the first order response to the system. Furthermore, these basis functions are multiplied with each other to produce the functions used for reproducing nonlinear responses.

A visualization of the Laguerre basis functions is shown in Figure 3. The basis functions are then scaled with coefficient values that are fitted to provide the appropriate response when all functions are convolved with the input signal and summed. To capture nonlinear responses, the basis functions are cross multiplied with each other as described previously. These functions together correspond to one set of basis functions with one given decay value, *p*. Because of the complexities of receptor responses, two sets of basis functions are used with different decay values represented by *p*. The first set covers the general response of the system within a short time frame to capture the overall waveform. The other covers a much longer time frame and accounts for slower mechanisms, such as desensitization. We found that using two basis function sets yield better approximation of the dynamics seen in the original kinetic models. The *p*-values were determined via gradient descent to find the optimal decay values with the lowest absolute error while fitting the data. The fitting process is further elaborated later in the description on coefficient estimation.

Note that increasing the order exponentially increases the number of equations and coefficients used for the input-output model. Previous uses of the generalized Laguerre-Volterra model (Song et al., 2009b) have shown that models up to the 3rd order are generally sufficient for modeling most neural spiking activity. In this study the model uses 3rd order for low frequency presynaptic activity and 4th order for higher input frequencies.

#### Model estimation and validation

The IO receptor models were trained using a series of Poisson random interval train inputs and with the responses of the kinetic models of AMPA and NMDA receptors. These responses are the total conductance *g*_*AMPA*_ for the AMPA receptor, and the open state of the receptor *O*(*t*) for the NMDA receptor. The open state was chosen as the response to be modeled for the NMDA receptor to avoid complications that may arise with the Magnesium blockade; this blockade is thus factored in afterwards when calculating NMDA conductance. The input frequency used for training was either 2 Hz for 3rd order models, or a hybrid of 2 and 10 Hz frequency trains for both 3rd order and 4th order models. Poisson random impulse trains (RIT) are used because they provide broadband input in order to highlight nonlinearities and dynamics for a wide range of input patterns, rather than with events at fixed intervals. A total of 1000 input events was used for training for 3rd order models and 2000 input events for order 4th models (this ensured a broad spectrum of input was covered for both 2 and 10 Hz mean input frequencies). The number of Laguerre basis functions used for each set is 4 for the AMPAr 3rd order IO receptor model. The number of basis functions per set was reduced to 3 for the NMDAr model with cross terms and for 4th order models; for models with cross terms and higher order models, the number of coefficients needed to be estimated is much larger, therefore the number of basis functions per set was changed to reduce calculation time required.

The IO synapse model coefficients were estimated with the MATLAB simulation environment and the Control System toolbox. Training the model involved iterating over various decay constants *p* for each set to find optimal values, yielding the lowest absolute error between the actual response of the model and predicted response using the estimated coefficients and decay constants. Estimation of the coefficients was done by taking the inverse function of the basis functions multiplied with the training data:

$$\begin{array}{l} c=\;y\cdot V^{-1}\left(t\right)\\ \hat{y}\left(t\right)=c\cdot V(t) \end{array}$$

Here, V(t) represents the matrix of all the basis functions (including both sets of basis functions with their given *p*-values) and their cross terms convolved with the input, x(t). This matrix is inverted using the pinv function in MATLAB, then multiplied with the training data to estimate the best fit coefficients for the IO model. The training estimate values are subtracted from the true training data set to determine the difference at each instant; these differences are then summed together to determine the absolute error of the training estimate given the *p*-values and the estimated coefficients. Finally, *p*-values are determined by gradient descent, choosing the *p*-values with the lowest absolute error when training to fit the data. The optimal *p*-values we obtained were 0.52 and 0.03 for the basis function sets associated with AMPAr, and 0.049 and 0.002 for the basis functions associated with NMDAr.

Validation of the optimal coefficients and decay constants was performed following the training procedure; it was done with a novel RIT input different from the input used for training. The length of the input signal for validation was set to 20 s. The average frequency of the input signal is generally 2 Hz unless otherwise specified. For validation, the normalized root mean square error (NRMSE) was calculated as shown in the following manner:

$$\begin{array}{l} NRSME={\left(\frac{\sum_{i=1}^{N}{\left(y\left(t_{i}\right)-ŷ\left(t_{i}\right)\right)}^{2}}{\sum_{i=1}^{N}y{\left(t_{i}\right)}^{2}}\right)}^{\frac{1}{2}} \end{array}$$

where y represents the expected response of the EONS synapse model, ŷ is the predicted response generated by the IO synapse model, and N is the total length of the response. In the responses the baseline is set as 0; as such, the error analysis presented here tracks mainly the differences in amplitude values and nonlinearities between the actual and estimated models. Estimated coefficients were stored in a file and then loaded into the NEURON simulation environment (Hines and Carnevale, 1997).

#### Implementation in the NEURON environment

The IO synapse model structure was implemented in NEURON with the use of module (mod) files. The module files accept the decay values and the coefficients as parameters. Because the number of basis function equations and coefficients differs between models of different orders, separate module files were made for different order models. In general, 3rd order models were used with 2 Hz Poisson random interval train events to simulate synaptic activity. The 4th order model was tested when simulating with higher frequency inputs and compared to the 3rd order model. In our models, the third order model requires 68 coefficient values whereas 4th order models use 209 coefficients—thus, the number of inputs to the module files is different. As a result, the basic structure of the IO synapse model, which is dependent on model order, is described in the module file. Notably, the module file was set to have a memory window of 2 s, keeping all input events triggered within the last 2 s of the current time point in memory for calculation of the IO synapse model responses. The width of this 2 s memory window was chosen as there were no significant contributions to the responses for events that take place more than 2 s prior to the current time point.

#### Simulation and model configuration

To test the IO synapse model in a cell simulation, the CA1 pyramidal cell model proposed by Jarsky (Jarsky et al., 2005) is used as a template. Synapse locations are randomly generated on the apical dendrite of the cell. Unless otherwise indicated, simulation input trains consist of Poisson random interval trains having a mean frequency of 2 Hz and the number of synapses is 16. NEURON simulations with the use of the EONS synapse model were run on cluster nodes with dual quad-core Intel Xeon 2.3 GHz processors with 16 Gb RAM. IO synapse model simulations were conducted with a single Fedora-based computer with Intel quad-core 2.67 GHz processor and 8 Gb RAM. All results were obtained with 20 s of simulated time.

## Results

### The IO synapse model accurately reproduces nonlinear dynamics seen in the parametric EONS synapse model

**Figure 4:**
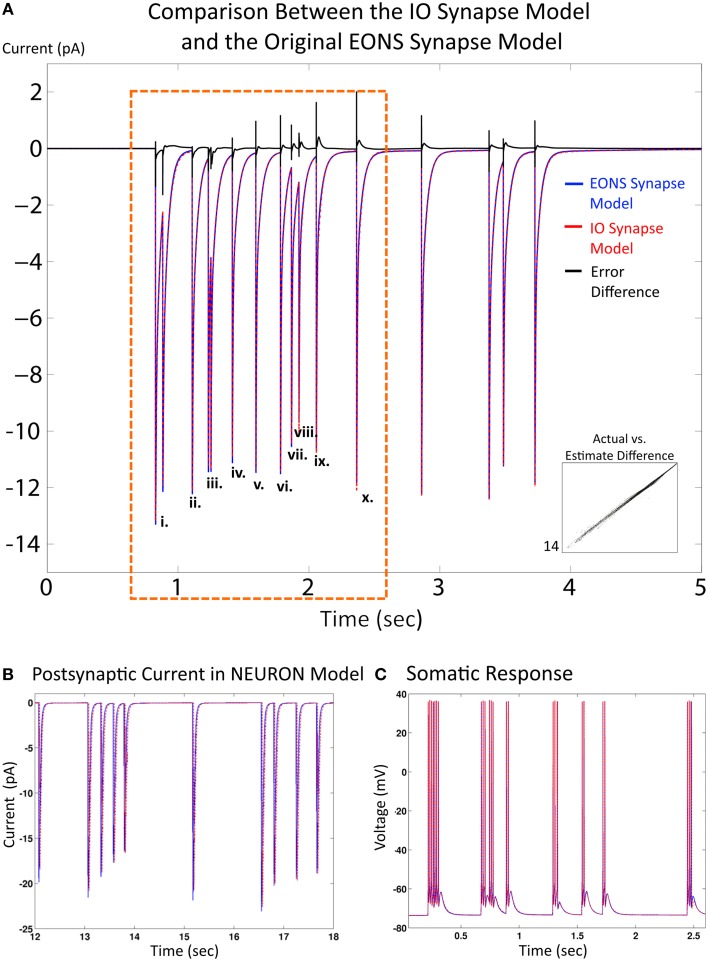
**Comparison between the IO synapse model and the original EONS synapse model. (A)** Expected EPSC response from the original EONS synapse model (blue) and the IO synapse model (red, dashed). The error difference between the two traces is plotted in black, to allow for comparison on when the two models diverge. Both simulations were run in the single synapse configuration and in voltage-clamped conditions. (i) through (x) designate individual or double responses to events within the first 2 s of presynaptic activity - the NRMSE of each response is presented in Table 1. Scatterplot on bottom right shows direct comparison of y-values (current) between the two models, where each point represents a different time point in the results. Results are shown to be nearly identical to each other, with only minor differences as shown in the error comparison and scatterplot. **(B)** Response from the EONS synapse model (blue) and the IO synapse model (red) when connected to a neuron model within the NEURON simulation environment. **(C)** Somatic response in a neuron model using the EONS synapse model (blue) and the IO synapse model (Red, dotted) within the NEURON simulation environment. In the simulation, stochastic vesicle release was disabled for consistency and all synapses fired in response to a pre-synaptic event. Both responses are nearly identical. The minor differences noted in the synaptic current as shown in the other comparisons do not significantly affect the response of the postsynaptic cell.

Results and simulation times for both the IO synapse model and the original platform were compared under the same simulation conditions (20 s simulated time, 2 Hz Poisson random interval train input) to evaluate performance of the IO synapse model in terms of accuracy and speed. For the original platform, simulations were run in Java, the native language code of the EONS/RHENOMS synaptic platform. The IO synapse model was prototyped first in MATLAB, then ported to the NEURON simulation environment. Two scenarios were explored in these studies. For the first one, synapse models were simulated alone (independently from their connected neuron) and compared for speed and accuracy (i.e., in a simulated voltage-clamped condition). The NRMS error was 3.3%, making the IO synapse model a reliable alternative to the original platform. Further investigation highlights the differences in error for individual events (or pairs of events if they are close to each other) for the first 2 s with presynaptic activity. This duration was chosen as it equals the memory window of the IO synapse model. Consequently, the response of the IO synapse model takes into account the nonlinear interactions induced by previous events that are within a 2 s timeframe of the current time point. Table 1 shows the NRMS error for the events indicated as shown in Figure 4A. Here, we see that some error is present even in the first few events without any previous activity. This error would indicate that lower order nonlinearities are being partly compensated in the model to better fit higher order linearities. The error overall remains fairly constant at around 3% until reaching the 10th event, where the last three events indicated are shown to deviate more significantly. Furthermore the direction of the error is different in comparison between the first few events and the following, as seen in the error difference chart on the top of Figure 3. In the first few events, the IO synapse model has more under-estimate error, whereas in the last few events observed, the error consisted in over-estimation of the output. Such analysis shows that the IO synapse model is accurate not only according to the overall RMS error, but even high order nonlinearities are well fitted in the model. This results in some compensation from the lower order nonlinear interactions, but in return responses that must consider up to 10 events in the past are still described accurately by the IO synapse model.

**Table 1:** **NRMSE comparison between the EONS synapse model and the IO synapse model**.

Synapse model alone, total	3.3%
i. 1st + 2nd event	3.03%
ii. 3rd event	2.87%
iii. 4th + 5th event	3.77%
iv. 6th event	3.07%
v. 7th event	2.1%
vi. 8th event	2.2%
vii. 9th event	2.49%
viii. 10th event	4.47%
ix. 11th event	6.82%
x. 12th event	5.08%
Synapse model with NEURON	5.66%

*i through x refers to the error comparisons of the event responses as shown in Figure 4. All events indicated are within a window of 2 s, which corresponds to the memory window of the IO synapse model. Hence the response at x. considers all nonlinear contributions of events preceding it*.

In the second scenario, we placed 16 synapses on a CA1 pyramidal neuron model in the exact same configuration for both the EONS and the IO synapse models. The original parametric EONS-NEURON framework used a communication protocol that ran MPI for interfacing multiple nodes processing the EONS synapse model to communicate with the NEURON simulator; for details on the NEURON simulation environment, please see (Hines and Carnevale, 1997). At every time point in the simulation, instances of the EONS synapse model calculate postsynaptic current based on the postsynaptic potential of the neuron model from the previous time point. The neuron model then uses the postsynaptic currents from EONS to re-calculate the postsynaptic potentials at the locations of the synapses and at the soma. As such, there is always an error between the value calculated from the previous potential and its current value. To minimize this error, the difference between two successive time points must remain small. Furthermore, in the EONS synapse model, the neurotransmitter release model required a time step of 0.5 μs in order to accurately simulate the diffusion profile of the neurotransmitter release (Bouteiller et al., 2008). Using step sizes larger than 0.5 μs yielded an inaccurate representation of the neurotransmitter diffusion profile in the cleft. To ensure accurate representation during the training phase of the IO synapse model, we therefore simulated results from the EONS synapse model at a fixed time step of 0.5 μs. Additionally, it ensures all events are captured and errors as a result of desynchronization remain minimal. Results used for validation were also run at 0.5 μs for consistency. The IO synapse model was simulated directly within the NEURON environment and therefore the postsynaptic potential is accurately represented at every time point. The NRMS error comparison between the original EONS synapse model and the IO synapse model remained small at 5.66%, when compared to the original platform, and postsynaptic responses from both synapse models matched up relatively well, as shown in Figures 4B,C; comparison of somatic voltages yielded virtually identical profiles.

### Higher order IO synapse models more accurately replicate eons synapse model results when given randomized inputs with higher mean frequency

**Figure 5:**
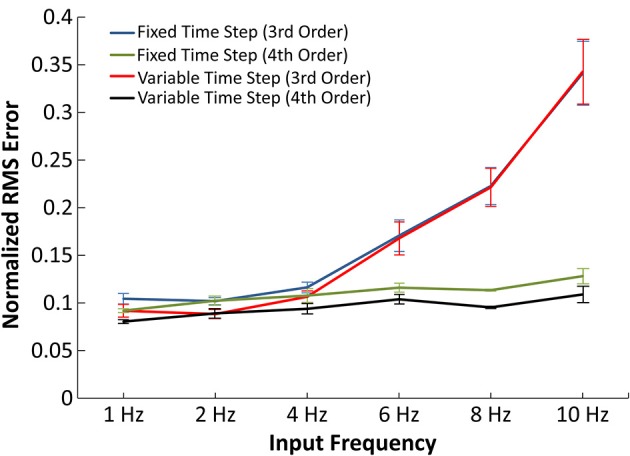
**Accuracy of the IO synapse model with various input frequencies**. The normalized RMS error is plotted for the 3rd order IO synapse model simulated at fixed (blue) and variable (red) time step simulations and for the 4th order IO synapse model simulated at fixed (green) and variable (purple) time step simulations. With the 3rd order model, the error noticeably increases at higher frequencies. The 4th order model yields constant error at all tested frequencies.

The 3rd order IO receptor models were trained with a random interval train input having a mean frequency of 2 Hz. This frequency value was chosen to reflect the typical firing rate of hippocampal CA1 and CA3 neurons (Berger et al., 1988a). However, the mean firing rate (MFR) can often vary widely within hippocampal pyramidal cells, even during the resting state (Ranck, 1973). We therefore opted to evaluate how the accuracy of the IO synapse model varied with different input frequencies. Thus, 3rd and 4th order IO synapse models were trained with randomized input events. The input events used for training consisted of a concatenation of random Poisson trains with a mean frequency of 2 and 10 Hz. The responses of the IO synapse models were then validated against the original EONS synapse model results for accuracy measurement. The input events for the validation tests consisted of Poisson randomized input events with a range of mean frequency values, as shown in Figure 5. IO synapse model simulations were conducted in both fixed time step and variable time step. Fixed time step was simulated with an interval of 0.1 ms; this corresponds to the bin size used for training the receptor models. Variable time step was used to further reduce calculation time for the IO synapse model. The method used was CVODE, an algorithm readily available in NEURON (Hines and Carnevale, 1997). The error for both simulations were compared with each other to assess whether there were discrepancies between fixed and variable time steps.

In Figure 5, the normalized RMS error was plotted as a function of average input frequency rate for 3rd and 4th order IO synapse models. In the presented results, the 3rd order IO synapse model was re-trained with a new set of input events in order to better capture the higher frequency nonlinearities. The training input events consisted of a hybrid of 2 and 10 Hz Poisson randomized interval trains: the first 500 events averaged a mean frequency of 2 Hz and the second 500 events averaged a mean frequency of 10 Hz, for a total 1000 input events. The IO synapse model was validated with simulations using input frequencies of 1, 2, 4, 6, 8, and 10 Hz. In validation, simulations at higher frequencies resulted in higher error for the 3rd order IO synapse model when compared with results from the EONS synapse model, with normalized mean square error (NRMSE) of up to 35% in simulations with 10 Hz input frequency. At lower frequencies (1–4 Hz) the error remained around 10%.

Higher frequencies are commonly associated with more nonlinear behavior. In order to more accurately account for such nonlinearities, we implemented an IO synapse model utilizing 4th order Volterra functional power series. The 4th order IO synapse model was trained and validated similarly to the 3rd order IO synapse model; the only difference was the number of input events, which was increased from 1000 to 2000 events to better estimate the large number of coefficients. The 4th order model was found to have an error difference of 10% across the frequency spectrum when compared to the EONS synapse model and thus captures nonlinearities associated with higher frequencies more accurately (up to 25% more accurate with 10 Hz input) than the 3rd order IO synapse model.

### Computational complexity of kinetic receptor models are reduced in IO synapse models within NEURON

A large portion of the calculation time required for the EONS synapse model is due to the data exchange process between the EONS synapse model and the neuron model (Allam et al., 2012). This, however, does not clearly establish whether there is benefit from using the Volterra functional power series over kinetic models. To investigate this, the two postsynaptic receptor models, AMPAr (Robert and Howe, 2003) and NMDAr (Ambert et al., 2010) were re-implemented in NEURON using kinetic modeling schematics (Carnevale and Hines, 2006). The kinetic models are functionally identical to the models used in the EONS synapse model, but now implemented in the NEURON environment using NEURON integrated ODE solvers. These two kinetic models were used together and their output currents summed to determine postsynaptic current from the synapses in the neuron model. This configuration allows for a direct comparison of simulation times between the EONS synapse model and the IO synapse model as the only difference lies in structure between the two model types. The Jarsky hippocampal CA1 pyramidal cell model was used for all simulations (Jarsky et al., 2005). Additional simulations were conducted with a single compartment Izhikevich model with similar results; these results were further analyzed in a manuscript yet to be published. All simulations were run using adaptive time step methods, and total number of steps are also presented to demonstrate difference in the number of steps required per simulation.

In the first condition, synapse weights were calibrated to reflect physiological conditions leading to postsynaptic neuron firing (Table 2). Under these conditions, the IO synapse model required 617 s of simulation time with 67,319 simulation steps, while the kinetic models required 1090 s and 107,640 steps. In the second condition, synapse weights were reduced to 0. This condition was chosen to investigate the simulation time required with minimal neuron model computation, thus reflecting more of the contributions of the synapses to the simulation benchmarks. Calculation of the compartments within the neuron model still take place, however as the inputs to the neuron is set to 0, calculations by the neuron model should have minimal influence on the simulation time. Under these conditions, the neuron using IO synapse models required 3.7 s of simulation time with 417 steps, while the one using kinetic models needed 589 s and 40,760 steps. In both cases, the IO synapse model outperforms the kinetic models and requires less steps for calculation. Notably, the speedup is more significant in simulations in which the computational contribution of the neuron model is minimal (i.e., when simulation times are used to calculate synaptic models only).

**Table 2:** **Simulation time and number of steps required to simulate the original kinetic models vs. IO synapse model within NEURON, as well as the speedup between the two conditions**.

	**Action potential fired**		**No action potential**	
Kinetic models	1090 s	107,640 steps	589 s	40,760 steps
IO Synapse model	617 s	67,319 steps	3.7 s	417 steps
Speedup	1.77x		159x	

*Kinetic models in general require more steps to calculate thus leading to longer simulations in simulations utilizing adaptive time step algorithms*.

Several points can be made about the results of these simulations. First, regardless of whether the kinetic models or the IO synapse model was used, the neuron model independently of the synaptic model used can take up a significant portion of simulation time depending on its complexity. Reducing the synaptic weight to 0 minimizes the neuron model's computational weight to the simulation, thereby emphasizing the synaptic component. Furthermore, all simulations conducted in this part of the study were simulated within NEURON under adaptive time step conditions. This results in a direct comparison between the kinetic models and the IO synapse model based on the Volterra functional power series. Differences in simulation time and number of steps seen in the results are therefore directly indicative of the difference in modeling methodology. Notably, the relative speedup between the IO synapse model and the kinetic models is shown to be significantly larger in simulations with the synaptic weights being set to 0. This result suggests that (1) calculation of the changes in potential in the neuron model compartments requires a significant amount of simulation time, and (2) for synapse activity alone, kinetic models require much more steps, thus resulting in longer simulation times compared to the IO synapse model. This is likely because kinetic models contain rate equations which require previous time points to calculate the state values. In contrast, the Volterra functional power series models the conductances of the receptors and accounts for the nonlinearities analytically—as a result, the response of the IO synapse model does require past values to calculate present values.

**Figure 6:**
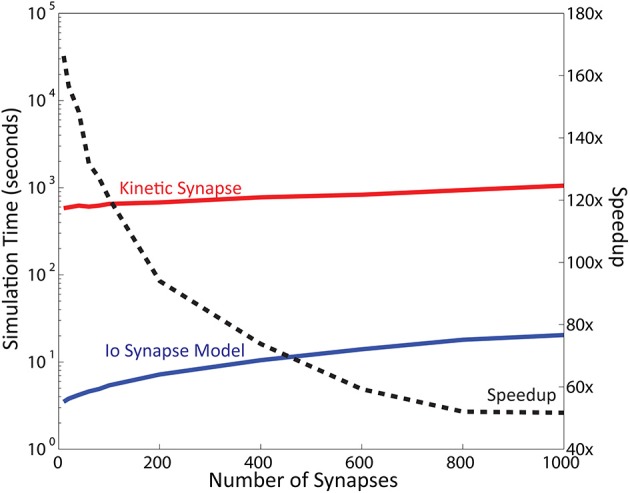
**Simulation Time varies as a function of the number of synapse instances**. Here, simulation time is represented in logarithmic scale. Computation time required for the kinetic synapse model is within the range of 10–20 min, while the computation time required for the IO synapse model ranges between 3 and 30 s. Dashed line represents the speedup of the IO synapse model against the kinetic synapse model based on number of synapses. At low number of synapses, the speedup of the IO synapse model is highest at around 150x faster than the computation time required for the kinetic synapse model. The speedup is shown to be decreasing, but stabilizes at around 50x speedup in later values.

Of importance, the number of synapses modeled also impacts computational speed. Neurons have a large number of synapses: a typical pyramidal CA1 neuron has been reported to have up to 30,000 synapses (Megìas et al., 2001). The results reported in the previous sections were obtained with 16 synapses. To address this point we varied the number of synapses distributed on the neuron and recorded the simulation times (Figure 6; also see Supplementary Figure 1 for more details). For these simulations the computational contributions of the neuron model were minimized by reducing synaptic weight to 0. The results indicated that the neuron comprised of IO synapse model retains a total calculation time of less than 1 min even in simulations with up to 1000 synapses. Meanwhile, the calculation time required for the kinetic models ranges from 10 min with 10 synapses, to 18 min with 1000 synapses. The number of steps remained almost constant for both models: 400 steps for the IO model, and 40,000 steps for the kinetic model. Measuring the approximate speedup of the IO model in comparison to the kinetic model as a function of the number of synapses gives approximately a 150x speedup at 10 synapses, number which decreases as the number of synapses increases, to finally stabilize at about 50x speedup for 1000 synapses. Additional tests performed at up to 5000 synapses confirm that beyond 1000 synapses, the speedup of the IO synapse model remains around the same at around 50x.

## Discussion

Utilization of detailed models, such as Markov kinetic states receptor models, reflect the inherent mechanistic properties of the system of interest. Their utilization can shed some light on potential abnormalities and dysfunctions underlying pathological cases, as well as identify possible solutions to re-establish normal receptor function, thereby facilitating identification of new therapeutics. Although these models are designed to be as mechanistically close as possible to the physiological structures they represent, their computational complexity often restrict the extent of what may be simulated, thereby making large-scale simulations impractical. This consequently impedes the creation of a unified computational platform that bridges micro- to macroscale dynamics. Yet function and dysfunctions that appear in pathological cases often stem from modifications at the molecular level, giving rise to altered macroscopic levels of activity leading to the observed phenotype. It is therefore essential to determine how changes propagate from the molecular and synaptic levels up to the network level. In this article, we described the development and characteristics of an IO synapse model that extracts and successfully replicates the functional properties generated by detailed kinetic models, while significantly reducing computational complexity thereby enabling simulations at a larger scale.

To do so, training and calibration of the IO synapse model was done on a local scale, yielding parameters values for the Volterra functional power series to accurately describe the dynamics of the system with a low computational complexity. We assessed the accuracy of the model compared to its parametric counterpart, and determined the speed gain obtained. We then went on to demonstrate that large-scale simulations could be performed with a very large number of IO synapse models, number unreachable using traditional parametric models.

Synapse models routinely used in large scale simulations typically consist of linear exponential synapses (Roth and Rossum, 2000; Hendrickson et al., 2012). These synapses, while sufficient to replicate the global waveform of synaptic responses, are not detailed enough to replicate nonlinear behaviors or to have any application for studying molecular-level modifications or drug-target interactions. On the contrary, the IO synapse model may faithfully reproduce nonlinear dynamics under a wide range of conditions (e.g., pathological or drug-treated), with a computational cost nearly equivalent to the exponential synapse.

The Volterra functional power series was chosen as the framework for the IO synapse model for its capability to capture and fit nonlinear dynamics. In the current study, we demonstrated that by capturing nonlinearities up to the 4th order, the IO synapse model was able to adequately replicate synapse dynamics with input trains of varying frequency, ranging from an average of 1 to 10 Hz. Future renditions of the IO synapse model may include more complex models with higher nonlinear dynamics (e.g., functional contribution of metabotropic receptor). The methodology described in this study may not be sufficient for such models. Ongoing studies have therefore been initiated to further build on the Volterra functional power series, such as using maximum-likelihood method for estimating systems with binary output (Berger et al., 2010); using the Poisson-Volterra model where the output can be represented with Dirac functions weighted by amplitude of the postsynaptic responses (Song et al., 2009b); including sparsity to reduce the number of required coefficients (Song et al., 2013); and even accounting for non-stationary behavior through stochastic state point process filters (Chan et al., 2009) to overcome potential limitations associated with using higher order Volterra representations and ensure their applicability to more general cases.

In our current study, we used the EONS synapse model to develop two separate IO receptor models—the AMPAr and NMDAr models. Initially, we first considered the implementation of a single IO synapse model for the postsynaptic component of the model (rather than one for each receptor type). Since an input-output model is based on input-output relationships it was presumed that all nonlinearities may be taken together. This would then require only one overall IO synapse model to be implemented, rather than multiple IO receptor models, thus presumably becoming more computationally efficient. However, several key factors influenced the choice of making the system more modular. First, the different receptors have different rise and decay characteristics. This had an impact on the overall estimation accuracy and the dynamics were not properly captured due to different decay rates (results not shown). Consequently, the decision to use separate input-output models for each ionotropic receptor allowed for each of the models to be more adequately calibrated, yielding more accurate results. If the postsynaptic response was represented by a single input-output model, the rise and decay rate of the input-output model would need to be averaged out between the two receptor models that it represents. Another reason for separating receptor models is the existence of specific characteristics associated with different types of receptors. The magnesium blockade is a clear example of such model-specific characteristics; here, using a separate model allows for the magnesium blockade factor to be separately accounted for in the NMDAr IO model only. If the entire synapse model was represented by only one input-output model, it would not be possible to associate the magnesium blockade effect with only NMDAr. Consequently, a more complex multi-input model would be required.

When considering MSM, the complexity of a kinetic model can vary significantly depending on its states and mechanisms. For example, ionotropic receptor models can be represented with only a few states and equations (Robert and Howe, 2003; Ambert et al., 2010), but large systems with intricate pathways may require significantly more states and equations to model, as is the case of the metabotropic glutamate receptor (Doi et al., 2005; Greget et al., 2011). The IO synapse model circumvents this issue by capturing the input-output relationships of the kinetic models. This means that no matter how intricate the original model may be, the IO synapse model will attempt to capture and replicate the outputs of the model using the same functional structure (sets of basis functions and coefficients) and consequently with the same computational complexity. This can facilitate integration of a large number of microscopic components with less concern for growing complexities, as would be the case for kinetic models with high numbers of states and rate equations. Furthermore, input-output modeling can even be extended into macroscopic levels, e.g., to reduce neuron models to a set of Volterra functions. Such methods could provide a way to extend multi-scale modeling even further, modeling even larger neuronal ensembles.

Notably, one limitation of our IO synapse model is that its input-output reference data is captured from a parametric model, which may not capture the full dynamics of the biological element. Ideally, the IO synapse models would be trained using experimental results. However, individual synapses and their dynamics are particularly difficult to measure, making it difficult to extract long, stable, and consistent sets of data from synapses. In this case, kinetic models may be better suited for initial calibration, which are based on experimental studies of individual synaptic components such as receptors isolated in expression systems (e.g., oocytes). The results of the calibrated kinetic models can then be used to train the input-output models for multi-scale and large-scale application. Furthermore, experimental techniques may be perfected in the future to allow direct calibration of the IO synapse models, which can also be used to further refine mechanistic (kinetic) models, to subsequently provide further insights into mechanistic functions. Such dual mechanistic and non-parametric approach may be able to further refine simulation predictions at the single and multi-scale levels while further extending the range of complex nonlinear dynamics modeled all the way up to large-scale systems, providing further insights into the complex physiological mechanisms taking place in the nervous system during normal function or pathological dysfunctions, and consequently help identify efficacious therapies to alleviate them.

### Conflict of interest statement

The authors declare that the research was conducted in the absence of any commercial or financial relationships that could be construed as a potential conflict of interest.

## Supplementary material

Supplementary Figure 1 **A linear representation of Figure 6**. The left axis represents the simulation time range for the IO synapse model while the right axis presents the range for the kinetic synapse model. Both models increase linearly; however, for the overhead simulation time that is irrelevant to the number of synapses, the kinetic synapse model has a much higher overhead than the IO synapse model.
