# Supplementary figures and images for: Volterra representation enables modeling of complex synaptic nonlinear dynamics in large-scale simulations
Source: Front Comput Neurosci. 2015 Sep 17;9:112. doi: 10.3389/fncom.2015.00112 (PMC4585022; doi:10.3389/fncom.2015.00112)

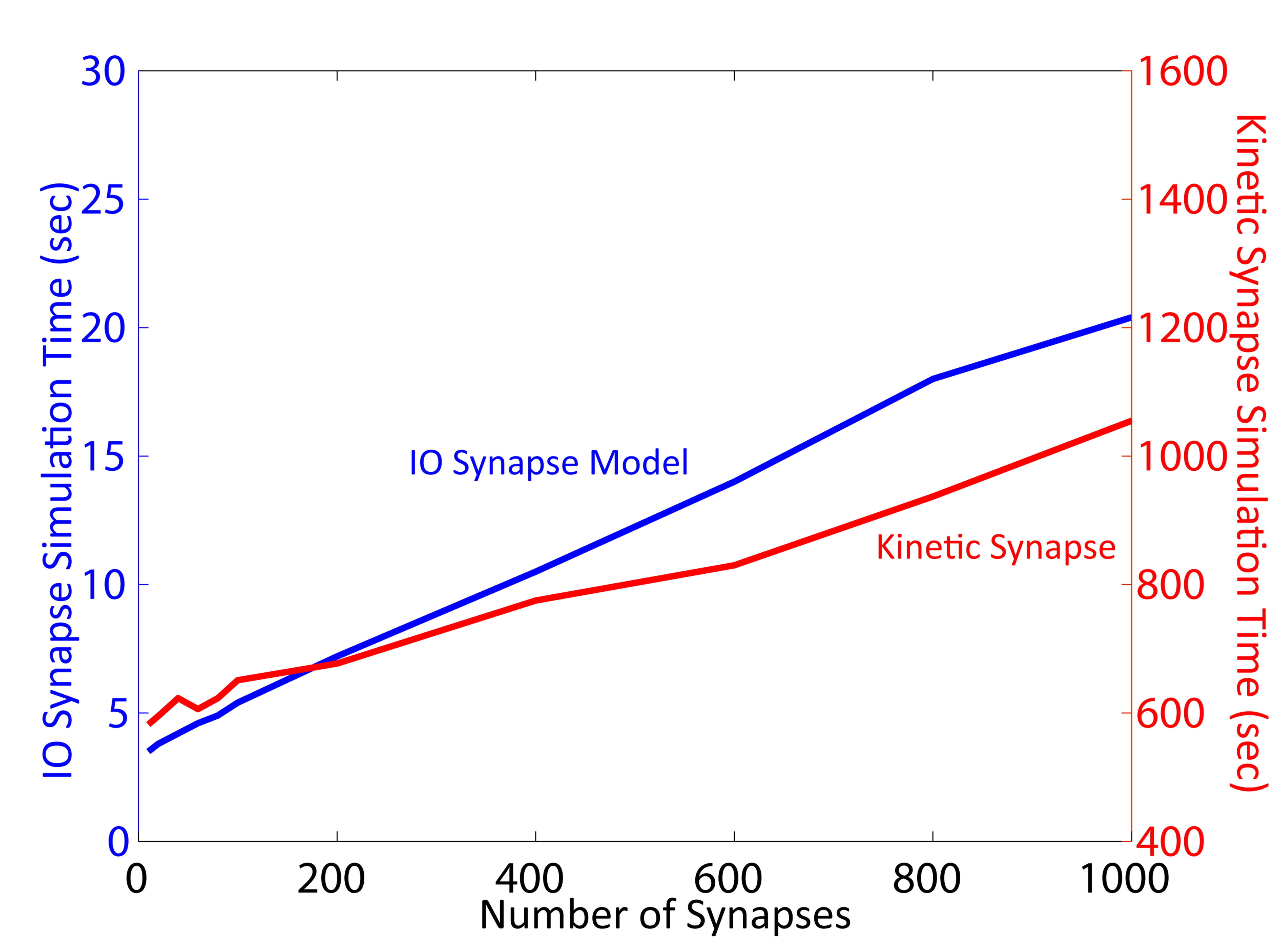

Supplement: Supplementary Figure 1 — A linear representation of Figure 6. The left axis represents the simulation time range for the IO synapse model while the right axis presents the range for the kinetic synapse model. Both models increase linearly; however, for the overhead simulation time that is irrelevant to the number of synapses, the kinetic synapse model has a much higher overhead than the IO synapse model. [file Image1.TIF]
